# Match running performance preceding scoring and conceding a goal in men’s professional soccer

**DOI:** 10.1038/s41598-024-63785-3

**Published:** 2024-06-05

**Authors:** Marek Konefał, Błażej Szmigiel, Bogdan Bochenek, Ryland Morgans, Piotr Żmijewski

**Affiliations:** 1 Department of Human Motor Skills, Wrocław University of Health and Sport Sciences, Paderewskiego 35, 51-612 Wrocław, Poland; 2 Wrocław University of Science and Technology, Wybrzeże Stanisława Wyspiańskiego 27, 50-370 Wrocław, Poland; 3 School of Sport and Health Sciences, Cardiff Metropolitan University, Cardiff, UK; 4 Department of Biomedical Sciences, Józef Piłsudski University of Physical Education in Warsaw, Marymoncka 34, 00-968 Warsaw, Poland

**Keywords:** Football, Time-motion analysis, HSR distance, Sprinting distance, Polish Ekstraklasa, Contextual variables, Psychology and behaviour, Physiology, Health occupations

## Abstract

This study aimed to investigate the potential differences in the match running performance of professional soccer players 5 min. before scoring and conceding a goal in the Polish Ekstraklasa. The sample consisted of 278 matches with 570 goals scored during official matches of the 2022/23 Polish Ekstraklasa season. All data was collected utilising the computerised multiple-camera optical tracking system TRACAB. Total distance covered (TD), standing distance (StD; < 0.72 km h^−1^), walking distance (WD; 0.73–7.2 km h^−1^), jogging distance (JG; 7.21–14.4 km h^−1^), running distance (RD; 14.41–19.8 km h^−1^), high-speed running distance (HSR; 19.81–25.2 km h^−1^) and sprinting distance (SprD; > 25.2 km h^−1^) were analysed in 5-min intervals prior to a goal scored for both teams. The employed linear mixed models showed that all examined match-running performance metrics were higher in teams that scored a goal compared to teams that conceded a goal. Within 5 min before scoring a goal in Polish Ekstraklasa matches, the scoring team produced significantly greater TD (∆ 95%CI 256.8–300.4 m; p = 0.001), WD (∆ 95%CI 52.3–95.8 m; p = 0.001), JG (∆ 95%CI 100.5–144.1 m; p = 0.001) and RD (∆ 95%CI 16.2–59.8 m; p = 0.001) compared to the conceding team, although no differences were found for HSR and SprD. These results demonstrate the enhanced identification potential of key physical performance factors influencing goal scoring in the Polish Ekstraklasa, thereby optimising the training process and improving overall performance. To enhance the effectiveness of soccer training, coaching and performance staff should consider this study's findings, that indicate an increase in the volume of medium- and low-intensity running efforts preceding a goal.

## Introduction

It is well evidenced that match running performance depends on context-related variables^1,2^. Recent time-motion analysis studies have provided a wealth of information examining the impact of match status, match outcome or quality of opposition on the physical performance of professional soccer players^3–5^. However, the inter-relationships between these variables, while well documented, are not always univocal. It is recognised that typically, high-ranked teams cover a greater total distance and sprint distance than lower-ranked teams^6^. However, on the other hand, other research indicates that following conceding a goal, teams display a high level of both total distance covered and high-speed running distance^7^. Moreover, it should be noted that the highest level of elite soccer match-play is quantified by an increased effort when in a defensive phase of the match, and a decreased effort during offensive match patterns^8^. This notion has been confirmed by Jerome et al.^9^, highlighting the lower physical intensity when the team has ball possession, but greater physical intensity when out of possession and attempting to regain the ball. Although, it is worth noting that statistics on the most effective ball possession strategies (fast attack, positional attack) may differ between leagues^10^.

The inter-relation between physical performance and match status evolution has been identified^1,11^. As soccer is a low-scoring game, the team that is winning for most of the match playing time has no guarantee of obtaining a positive final result. For example, the team may lose a goal or goals during the latter stages of the match (last 15-min period) and thus lose the match. For this reason, researchers noted that the final match result depends on short moments within the game where the team scores or concedes a goal. These moments are contextually extremely important and worthy of detailed analysis. There are several studies describing the match running performance concerning short phases of match status^12^ and a physical “pacing pattern” employed by the team^13^. Also, concepts that consider specific phases of the match (changing or maintaining the match status)^7^ have recently been examined, although it is still not well reported on how physical performance changes immediately before scoring or conceding a goal. Previous research has established that following intense efforts, match running performance within a 5-min period may be diminished, even among elite players. Hence, tactical and motor behaviours of a team in the 5 min preceding the creation of an attacking advantage resulting in a shot on goal may warrant further investigation^14,15^.

Knowledge regarding the level of the match running performance of professional soccer teams before scoring a goal can provide coaches with information on team pacing behaviours and gaining a competitive advantage over the opponent and contribute to the practical preparation of their teams for such situations. Also, knowledge concerning the level of match running performance prior to conceding a goal can be used to increase or modify the intensity of player movement in order to avoid conceding goals. Therefore, this study aimed to investigate the potential differences in the match running performance of professional soccer players 5 min before scoring and conceding a goal in the Polish Ekstraklasa. Based on the above-mentioned studies, the study hypothesis was that the match running performance before a goal may differ significantly between the scoring team and the conceding team, and the differences depend on the intensity and type of activity.

## Materials and methods

### Study design

Utilising the computerised tracking system TRACAB, the match running performance of teams competing in official league matches of the Polish Ekstraklasa (the highest level of competition in Poland) during the 2022/23 season was recorded. Match data was then imported from the system, including splitting the entire match into 5-min time intervals. Following, the exact moments of goals scored during the analysed matches were determined. The exact values of the analysed match running performance in the 5 min before scoring or conceding a goal were determined proportionally employing 5-min time intervals recorded by the computerised tracking system. As a result, the analysed match running performance data obtained by the team that scored and the team that conceded the goal were compared. The results show differences in match running performance between teams that scored and conceded the goal.

### Sample

The whole season of the Polish Ekstraklasa 2022/23 consisted of 306 matches. The final sample consisted of 278 official league matches and 570 goals. Considering the effect of dismissals in match running performance^16^, 20 matches that involved a player dismissal (i.e., red card) were not included in the analysis. Furthermore, a total of eight matches were not available from the database.

In order to guarantee player and team confidentiality, all data were anonymised in accordance with the Declaration of Helsinki. Wroclaw University of Health and Sports Sciences Ethical Review Board waived the need of informed consent. The study was conducted according to the guidelines approved by the Wroclaw University of Health and Sports Sciences Ethical Review Board (agreement number: 12/2021).

### Variables and procedures

Match running performance data was collected utilising the computerised multiple-camera optical tracking system TRACAB^®^ (ChryronHego VID, New York, NY) with a 25 Hz sampling frequency. The validity and reliability of this video-tracking system have been established^17^. In accordance with previous research, match running performance was classified into the following categories: total distance covered (TD), standing distance (StD; < 0.72 km h^−1^), walking distance (WD; 0.73–7.2 km h^−1^), jogging distance (JG; 7.21–14.4 km h^−1^), running distance (RD; 14.41–19.8 km h^−1^), high-speed running distance (HSR; 19.81–25.2 km h^−1^) and sprinting distance (SprD; > 25.2 km h^−1^)^18^. Match running performance were analysed in 5-min intervals prior to a goal scored for both teams.

### Statistical analysis

In this study, linear mixed models were employed to predict trends in players' physical performance differences associated with scoring and conceding goals. By using fixed effects, common patterns in various performance metrics—collectively referred to as 'Parameters'—across all matches were analysed. 'Game' and 'Match week' are considered as random effects to cater for the uniqueness of each match and the period of the season, respectively. Specifically, the 'Game' random effect captures the characteristics of each match such as venue, criticality, and opposition quality, that might affect performance but are not the primary focus of this study. 'Match week', as another random effect, adjusts for the time-varying aspects of the season that could influence all teams, namely player form and fatigue as the season progresses.

All analyses were conducted using Python 3.9 language with the “statsmodels” library^19^. Models were structured to analyse the differences in physical running performance of professional soccer teams 5 min before scoring and conceding a goal as a function of various parameters and scoring teams. These factors were treated as fixed effects in the model. In addition, the model accounted for the influence of the game (as a proxy for situational factors like match location, status, and quality of opposition) and match week as random effects, to address the repeated measurements for each team.

Model formula was as follows:

$$ {\text{Performance}}\_{\text{Difference }}\sim {\text{ C}}\left( {{\text{Parameter}}} \right) \, + {\text{ C}}\left( {{\text{Scoring}}\_{\text{Team}}} \right) \, + \, \left( {{1}|{\text{Game}}} \right) \, + \, \left( {{1}|{\text{Matchweek}}} \right) $$

This formula enabled the exploration of how different parameters of physical performance and the scoring team influenced the performance difference, while accounting for the variability across different matches and match weeks. Significance was established at the p < 0.05 level. In Table 1 ‘z’ column stands for z-score, which measures how many standard deviations the estimated coefficient (effect of a variable) is away from 0. It's primarily used for hypothesis testing (to determine if the effect is statistically significant). A large absolute value of the z-score (above 3) indicates that the effect is unlikely to be due to random chance. An effect size is exactly equivalent to a z-score of a standard normal distribution^20^.

**Table 1:** Statistical differences in 5-min match running performance between scoring and conceding teams in the Polish Ekstraklasa.

Match running performance	Typical 5-min match periods (mean ± SD)	Scoring team running performance within 5-min (mean ± SD)	Conceding team running performance within 5-min (mean ± SD)	Modelled differences between scoring and conceding teams (mean)*	z	p >|z|
TD (m)	5486 ± 1088	5643 ± 1100	5325 ± 1110	299.6	25.1	**0.001**
StD (m)	72 ± 80	77 ± 83	57 ± 64	18.9	1.5	0.126
WD (m)	1958 ± 326	2001 ± 333	1916 ± 328	74.1	6.7	**0.001**
JG (m)	2097 ± 566	2161 ± 571	2017 ± 568	132.6	11.0	**0.001**
RD (m)	909 ± 271	939 ± 280	891 ± 275	42.2	3.4	**0.001**
HSR (m)	357 ± 131	367 ± 134	352 ± 133	12.2	0.8	0.401
SprD (m)	93 ± 65	97 ± 63	90 ± 64	4.8	0.4	0.684

TD: total distance covered; StD: standing distance (< 0.72 km h^−1^); WD: walking distance (0.73–7.2 km h^−1^); JG: jogging distance (7.21–14.4 km h^−1^); RD: running distance (14.41–19.8 km h^−1^), HSR: high-speed running distance (19.81–25.2 km h^−1^); SprD: sprinting distance (> 25.2 km h^−1^); * Mean differences between scoring and conceding teams was presented as adjusted value based on liner mixed model.

Significant values are in bold.

## Results

**Figure 1:**
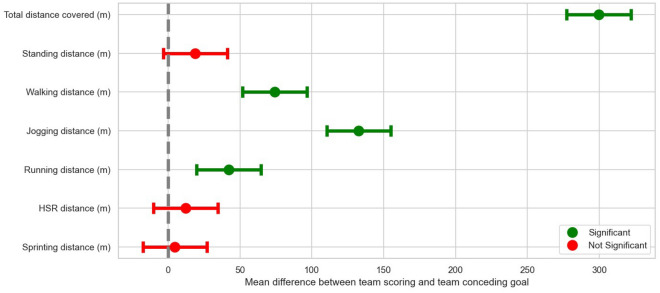
Differences in 5-min match running performance between scoring and conceding teams in the Polish Ekstraklasa (mean ± SD).

Table 1 and Fig. 1 display the differences in match running performance between teams that scored and conceded a goal in the Polish Ekstraklasa. During the last 5 min before the goal was scored, higher values of all analysed match running performances were recorded by teams that scored a goal compared to teams that conceded a goal. Significant differences in the teams that scored a goal were observed in total distance covered (∆ 95%CI 256.8–300.4 m; p = 0.001), walking distance (∆ 95%CI 52.3–95.8 m; p = 0.001), jogging distance (∆ 95%CI 100.5–144.1 m; p = 0.001) and running distance (∆ 95%CI 16.2–59.8 m; p = 0.001). However, no significant differences were found in standing distance (∆ 95%CI − 4.8 to 38.8 m; p = 0.126), high-speed running distance (∆ 95%CI − 12.5 to 31.1 m; p = 0.401) and sprinting distance (∆ 95%CI − 17.3 to 26.3 m; p = 0.684)—Table 1, Fig. 1.

## Discussion

To the authors knowledge, this is the first study that investigated the potential differences in the match running performance of professional soccer players within fixed time periods before scoring and conceding a goal in the Polish Ekstraklasa. The main findings were that all running performance metrics exhibited greater distances in teams that scored a goal compared to teams that conceded a goal. Significant differences were noted in total distance covered, walking distance, jogging distance and running distance. Conversely, non-significant effects were observed in standing distance, high-speed running distance, and sprinting distance. The primary conclusion drawn from the study is that the significant difference between teams scoring and conceding a goal lies in the distance covered during low-to-moderate intensity match running performances. This finding may seem somewhat unexpected, contrasting with prior research where authors predominantly demonstrated a correlation between match score and high-intensity running performance^7,21^.

Previously, Lobo-Triviño et al.^21^ identified a factor that significantly contributed to team success in the second division of LaLiga. This factor demonstrated positive relationships with various match running performance variables, such as total distance covered, sprint distance, and sprinting acceleration when the opponent team possesses the ball. Additionally, it showed associations with technical-tactical variables, including fouls received, tackles, and shots inside the box. Although, these researchers did not analyse shorter time periods (5 min or less) within the match. However, it must be recognised that high-intensity efforts are an important element of modern and effective soccer match-play and its contribution increases at the elite level, 80–90% of playing time, players move at a low-intensity^22^. A relatively large number of high-intensity actions requires adequately long recovery periods, during which players are attempting to maintain effective activity. Walking, jogging and running is an effective movement pattern that allows for faster recovery to occur between high-intensity efforts. As a result, increased frequency of high-intensity actions may imply modified team tactical and pacing behaviours and potentially increase the running distances covered at low- and moderate-intensity. Additionally, although low- and moderate-intensity match running performance does not guarantee a more effective fast attack, through organised movement of the entire team, it may be an effective strategy to employ during a positional attack^5,23^. In this context, it is worth emphasising that the present research included data for entire teams, and high-intensity efforts are characteristic of players playing in specific positions on the pitch^24^. Bradley and Noakes^12^ reported that attackers covered 15% and 54% more high-intensity running and sprinting in successful (win) matches compared unsuccessful (draw or loss), while central defenders covered 10–17% less high-intensity running during matches that were successful versus unsuccessful (lost or drawn). In contrast, Ponce-Bordón et al.^1^ research showed that total distance and distance covered above 21 km h^−1^ by offensive players significantly increased when teams were winning. Still, when it comes to defensive players such as central defenders and wing-backs, these metrics increased when teams were losing. Given these variables, it may be assumed that the running performance of the team that scored a goal may differ, e.g. depending on team formation or player's individual characteristics. Also, it has been shown that playing position and match location can affect the number of high-intensity efforts more than the quality of the opposition in elite football players^3^. Considering the above-mentioned findings, the present authors suggest that potentially exciting future studies that investigate positional variations in running performance before scoring and conceding a goal are warranted.

Previously, Schulze et al.^25^ conducted a study aimed at investigating 1- and 5-min periods of player and team running behaviours prior to goal-scoring opportunities. When analysing 5-min intervals, Schulze et al.^25^ also observed that the attacking team covers a greater total distance before scoring a goal, with an average of 104.2 ± 5.0 m min^−1^ per player, compared to 83.9 ± 6.4 m min^−1^ per player for the defending team, representing a 24% difference. In our study, this difference was lower at around 6%. Perhaps the occurrence of this variation may be attributed to the level of sportsmanship among teams. A similar trend was not found when analysing high-intensity distance based on individualised thresholds (15.7 ± 1.8 m min^−1^ vs. 17.3 ± 1.5 m min^−1^ per player)^25^. Also in our study, significant differences for high-intensity running and sprinting distances were not observed. In the previously mentioned study^25^, it was noted that increased attacking effectiveness correlated with increased distance covered in both attackers aimed at out-manoeuvring opponents and defensive actions employed by players tracking the opposing movements. Counter-attacks, characterised by fewer defenders positioned behind the ball, were observed to exert greater physical demands compared to direct play and intricate attacks, resulting in a higher frequency of goals^25^. The study also emphasised the pivotal role of robust physical capacity, as evidenced by diminished physical output during the match and the 5-min period leading up to an attempt being associated with success. This highlights the paramount importance of implementing tactical and pacing strategies to optimise physical outputs as necessary^25^. Furthermore, the availability of space, whether generated during counter-attacks or created by attackers through heightened running output, emerges as a decisive factor in goal-scoring opportunities during open play. The present study did not examine these factors, although a large sample of 278 official league matches and 570 goals is advantageous, and based on data from the highest professional level in Poland. Furthermore, differing relationships withing elite soccer exist, often resulting from the influence of many external factors, and thus are recommendations and motivation for further search into these differences.

When discussing the present results, some study limitations must be considered. Firstly, the match running performance was analysed using standardised speed thresholds. In this sense, it is well established that the lack of speed threshold individualisation can generate large differences in quantifying distance covered at high-intensity^26^. Furthermore, the presentation of the match running performance does not include data concerning match duration or effective playing time^27,28^. However, our research compares teams playing in the same match, so the lack of significant differences in these variables may justify such an approach. Future research should also consider the time of the goal, e.g. the first, second and third 15-min periods of the first and second half, due to fatigue resulting from accumulated playing time. Furthermore, the current study neglects some important and frequent physical actions, such as accelerations and decelerations. Future studies that incorporate these variables and aim to validate the existing findings in other soccer leagues or tournaments may be worthwhile. Additionally, an effective assessment of soccer performance at the behavioural level should be considered, including both the diverse contextual variables, and the interactions within the match. Finally, the specific strategic variables adopted by teams based on the evolving needs at each moment of the game^29^ are also warranted.

## Conclusions

The results of the current research indicate that all examined metrics of match running performance were higher in teams that scored goals compared to teams that conceded goals. In the Polish Ekstraklasa, within 5 min before scoring a goal, teams covered significantly greater distance at low- and medium-intensity when compared to before conceding a goal. These findings suggest that pre-scoring and pre-conceding situations in soccer are characterised by different running behaviours. Therefore, coaches and performance staff should tailor within-game tactical strategies based on significant situational moments such as scoring or conceding a goal, to optimise player and team match-play performance.

## Practical application

The findings of this study offered insight into enhancing the identification of key physical performance factors affecting goal scoring in the Polish Ekstraklasa. To enhance the effectiveness of soccer training and match-play, coaching and performance staff should consider the present findings when evaluating team behaviours, that indicate an increase in the volume of medium- and low-intensity running efforts preceding a goal.

## Data Availability

The datasets used and analysed during the current study are available from the corresponding author (marek.konefal@awf.wroc.pl) on reasonable request.
