# Author Correction: Match running performance preceding scoring and conceding a goal in men’s professional soccer

**DOI:** 10.1038/s41598-024-65400-x

**Published:** 2024-06-24

**Authors:** Marek Konefał, Błażej Szmigiel, Bogdan Bochenek, Ryland Morgans, Piotr Żmijewski

**Affiliations:** 1 Department of Human Motor Skills, Wrocław University of Health and Sport Sciences, Paderewskiego 35, 51-612 Wrocław, Poland; 2 Wrocław University of Science and Technology, Wybrzeże Stanisława Wyspiańskiego 27, 50-370 Wrocław, Poland; 3 School of Sport and Health Sciences, Cardiff Metropolitan University, Cardiff, UK; 4 Department of Biomedical Sciences, Józef Piłsudski University of Physical Education in Warsaw, Marymoncka 34, 00-968 Warsaw, Poland

Correction to: *Scientific Reports* 10.1038/s41598-024-63785-3, published online 05 June 2024

The original version of this Article contained an error in Affiliation 2, which was incorrectly given as ‘Wrocław University of Environmental and Life Sciences, Wybrzeże Stanisława Wyspiańskiego 27, 50-370, Wrocław, Poland.’ The correct affiliation is listed below:

Wrocław University of Science and Technology, Wybrzeże Stanisława Wyspiańskiego 27, 50-370, Wrocław, Poland.

The original Article has been corrected.

